# Identification of ferroptosis-related genes in heart tissues of patients with hypertrophic cardiomyopathy

**DOI:** 10.1097/MD.0000000000041525

**Published:** 2025-02-28

**Authors:** Fang Huang, Shujuan Li, Ailei Zhang, Jihuai Zhao, Shaoqiang Zhang, Dongwei Liu, Wei Chen

**Affiliations:** a Department of Cardiology, Qingdao West Coast New Area People’s Hospital, Cardiovascular Internal Medicine, Qingdao, Shandong, China.

**Keywords:** diagnostic biomarkers, differentially expressed genes, ferroptosis, hypertrophic cardiomyopathy

## Abstract

**Background:**

This study aims to investigate the role of ferroptosis in hypertrophic cardiomyopathy (HCM), a genetic disorder characterized by abnormal thickening of the heart muscle. The objective is to identify differentially expressed genes associated with ferroptosis in HCM and understand the potential molecular mechanisms underlying the disease.

**Methods:**

Comprehensive genomic analysis was conducted to identify differentially expressed genes associated with ferroptosis in HCM. The analysis focused on TFRC, SCD, SLC2A1, EGR1, GDF15, SNCA, PLIN2, and NQO1 as hub genes regulating ferroptosis. Functional enrichment analysis was performed to uncover their involvement in pathways such as ferroptosis, ubiquinone biosynthesis, and HIF-1 signaling. In addition, immune cell infiltration patterns in HCM were explored, and associations between the hub genes and immune infiltration were identified.

**Results:**

The analysis revealed TFRC, SCD, SLC2A1, EGR1, GDF15, SNCA, PLIN2, and NQO1 as hub genes involved in the regulation of ferroptosis in HCM. Functional enrichment analysis indicated their contribution to key pathways related to ferroptosis, ubiquinone biosynthesis, and HIF-1 signaling. Furthermore, associations between the hub genes and immune infiltration in HCM were observed.

**Conclusion:**

This study provides valuable insights into the molecular basis of HCM by identifying differentially expressed genes associated with ferroptosis. The findings suggest potential molecular mechanisms underlying the development of HCM. These findings contribute to a better understanding of HCM and may pave the way for the development of targeted therapies and improved diagnostic approaches for this debilitating cardiac disorder.

## 1. Introduction

Hypertrophic cardiomyopathy (HCM) is a genetic disorder characterized by the abnormal thickening of the heart muscle, resulting in impaired cardiac function and increased risk of sudden cardiac death.^[1]^ Despite extensive research into the pathogenesis and treatment of HCM, many aspects of its underlying molecular mechanisms remain poorly understood. One potential avenue of investigation is the role of ferroptosis in the development and progression of HCM.

Ferroptosis is a recently discovered form of regulated cell death that is distinct from apoptosis, necrosis, and autophagy. It is characterized by the accumulation of toxic lipid peroxides, leading to cellular damage and death.^[2]^ Emerging evidence suggests that ferroptosis plays a crucial role in various diseases, including cancer, neurodegeneration, and ischemia/reperfusion injury.^[3]^ However, its involvement in cardiac pathologies, particularly HCM, remains largely unexplored.

The purpose of this study is to investigate the regulatory mechanisms of ferroptosis in HCM by performing a comprehensive genomic analysis. By identifying key genes, pathways, and regulatory networks associated with ferroptosis, we aim to enhance our understanding of the molecular basis of HCM and potentially identify novel therapeutic targets.

In this study, we employed transcriptomic data analysis to identify differentially expressed genes (DEGs) associated with ferroptosis in HCM. By comparing the gene expression profiles of heart tissues from HCM patients with those from healthy individuals, we aimed to identify key genes that are involved in the regulation of ferroptosis in HCM. The identification of ferroptosis-related genes in HCM could have significant clinical implications. It may contribute to the development of targeted therapies that modulate ferroptosis pathways, potentially mitigating disease progression and improving patient outcomes. Additionally, the identified genes may serve as potential diagnostic or prognostic biomarkers for HCM, aiding in the early detection and monitoring of the disease.

**Figure 1.**
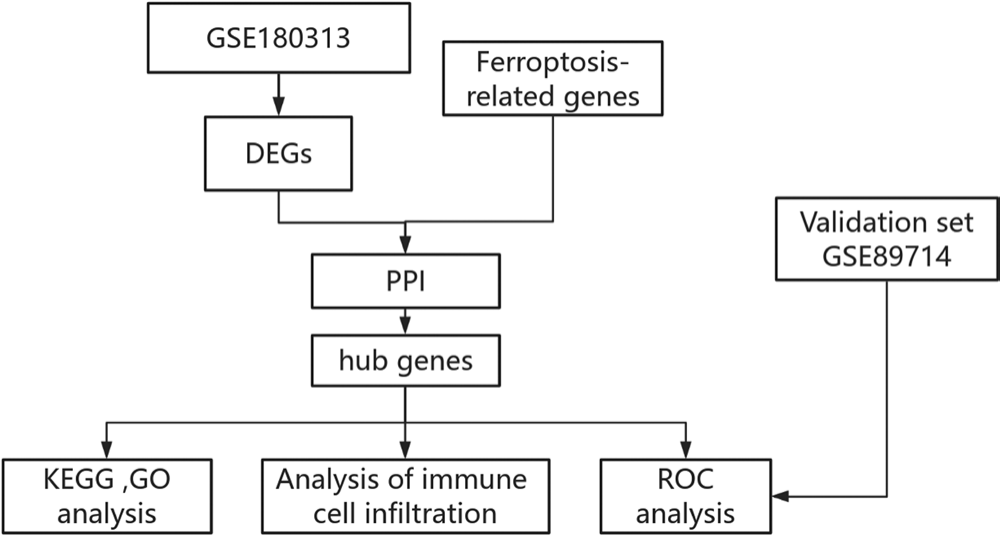
Flowchart of the approach to explore the mechanisms. DEGs = differentially expressed genes, GO = Gene Ontology, KEGG = Kyoto Encyclopedia of Genes and Genomes, PPI = protein–protein interaction, ROC = receiver operating characteristic curves.

This study aims to elucidate the regulatory mechanisms of ferroptosis in HCM through a comprehensive genomic analysis. The specific process was shown in Figure 1. By integrating multi-omics data, we hope to uncover key genes, pathways, and regulatory networks underlying the development and progression of HCM. The findings from this research may contribute to the development of targeted therapies and novel diagnostic approaches for this debilitating cardiac disorder.

## 2. Materials and methods

### 2.1. Data source and identification of DEGs

From the GEO database, microarray gene expression dataset GSE180313 pertaining to HCM were chosen. The GSE180313 dataset performed a comprehensive multi-omics profile of the molecular (transcripts, metabolites, and complex lipids), ultrastructural, and functional components of HCM energetics using myocardial samples from 27 HCM patients and 13 normal controls (donor hearts). The difference analysis of GSE180313 was performed using R Project, LIMMA and DESeq2 were used to standardization and differential analysis. The volcano plots and heatmaps of the DEGs in dataset were constructed using R, with a statistically significant cutoff value of |log_2_(FC)| > 1 and *P* < .05. We discovered 1054 genes by overlapping the DEGs from GSE180313. The GSE89714 dataset, which describes the differences in the heart tissue of 5 HCM patients and 4 normal hearts, was used as a validation set. Both male and female patients were included in dataset.

### 2.2. Hub genes identification and protein–protein interaction (PPI)

We used the “VennDiagram” package to intersect the genes obtained by DEGs and ferroptosis-related genes (Table S1, Supplemental Digital Content, http://links.lww.com/MD/O411),^[4]^ to further identify the HCM related hub genes. To integrate biomolecular interaction networks with high-throughput expression data and other molecular states, we obtained protein and functional interaction networks for the 26 combined hub genes from the STRING database (https://string-db.org/) and visualized them using Cytoscape.^[5]^ To investigate the hub gene network, we utilized the CytoHubb Plugin for Cytoscape. This plugin offers a variety of techniques for identifying critical nodes in biological networks and their connections to other genes. In our study, the top 10 hub genes were identified by employing diverse algorithms (closeness, MNC, edge percolated component [EPC], and betweenness ranking methods) to identify hub genes. The resulting network colored the hub nodes according to their relevance, with red representing the greatest score and yellow representing the lowest. We identified *TFRC*, *SCD*, *SLC2A1*, *EGR1*, *GDF15*, *SNCA*, *PLIN2*, and *NQO1* as the common interacting hub genes after completing a Venn diagram analysis on the top 10 hub genes from each ranking method. We extracted the expression of these 8 genes in GSE180313 and reanalyzed the expression data by *t* test, and found that the expression of 2 genes, *SCD* and *EGR1*, was not significantly different between the HCM and healthy groups. Then the rest of other 6 genes function in ferroptosis confirmed, won the final 4 hub genes, *SLC2A1*, *SNCA*, *PLIN2*, and *NQO1*.

### 2.3. Gene Ontology (GO) and Kyoto Encyclopedia of Genes and Genomes (KEGG) analysis

The combined set of 4 hub genes was analyzed for Gene Ontology (GO) enrichment and Kyoto Encyclopedia of Genes and Genomes (KEGG)^[6]^ pathway enrichment using the ShinyGO 0.77,^[7]^ which provides a user-friendly and visually appealing interface for enrichment analysis. The top 10 pathways were chosen for both the GO and KEGG studies based on their relevance. To identify the enriched pathways, a false discovery rate (FDR) value of <0.05 was used as the cutoff criterion.

### 2.4. Immune cell infiltration and correlation analysis

The immunological infiltrations of immune cells were analyzed using the CIBERSORT^[8]^ algorithm to investigate the immune microenvironment in HCM patients. B cells, T cells, NK cells, monocytes, macrophages, neutrophils and other immune cells were among the immune cells studied. The expression levels of 20 immune invading cells were depicted using box plots. The ggplot2 package was used to visualize the connection of different types of immune cells with SLC2A1, SNCA, PLIN2, and NQO1 using Spearman correlation.

### 2.5. Identification of diagnostic genes

Receiver operating characteristic curves (ROC) analysis was performed, and the AUCs were calculated using the pROC package in R software to determine the predicted values of the hub genes. Diagnostic genes were selected from the training set and validation set using the criterion of AUC > 0.700. To observe the dynamic changes in diagnostic genes in HCM, the expression levels and ROC curves of diagnostic genes in HCM and healthy patients were analyzed.

### 2.6. Cell culture

The H9C2 cells were cultivated in Dulbecco’s Modified Eagle Medium supplemented with 10% fetal bovine serum. The culture was maintained in a controlled environment at 37°C, with a 5% CO_2_ atmosphere.

### 2.7. Western blot analysis

Protein samples, containing 60 mg each, were separated by 12% SDS/PAGE and subsequently transferred onto polyvinylidene difluoride membranes. Following transfer, the membranes were incubated in a NaCl/Tris-T buffer (containing 10 mM Tris-Cl, pH 8.0, 150 mM NaCl, and 0.5% Tween-20) with 5% skim milk at 4°C overnight. After 3 washes with NaCl/Tris-T buffer for 10 minutes each, the membranes were exposed to the designated primary antibody against rat IgG coupled to horseradish peroxidase (Zymed Laboratories, San Francisco) for 1 hour. Immunodetection was facilitated using an enhanced chemiluminescence system (Roche Diagnostics, Germany) and visualized using a UVP BioSpectrum Imaging System (UVP Inc.). Densitometry analysis was applied to the resulting images. Specific antibodies included: rabbit anti-phosphorylated STAT3 (1:600, Sangon, China). GAPDH protein was utilized as a loading control in the Western blot analysis for quantitative purposes.

### 2.8. Statistical analysis

R 4.2.0 software and GraphPad Prism (version 8.0.1) were employed in this research. Data are presented as the mean ± SD, and comparisons between groups were performed using an unpaired Student’s *t* test. ROCs were used to evaluate AUCs and predictive abilities. A *P* value of <.05 was considered statistically significant.

## 3. Results

### 3.1. Identification of DEGs and ferroptosis-related differential genes in HCM

**Figure 2.**
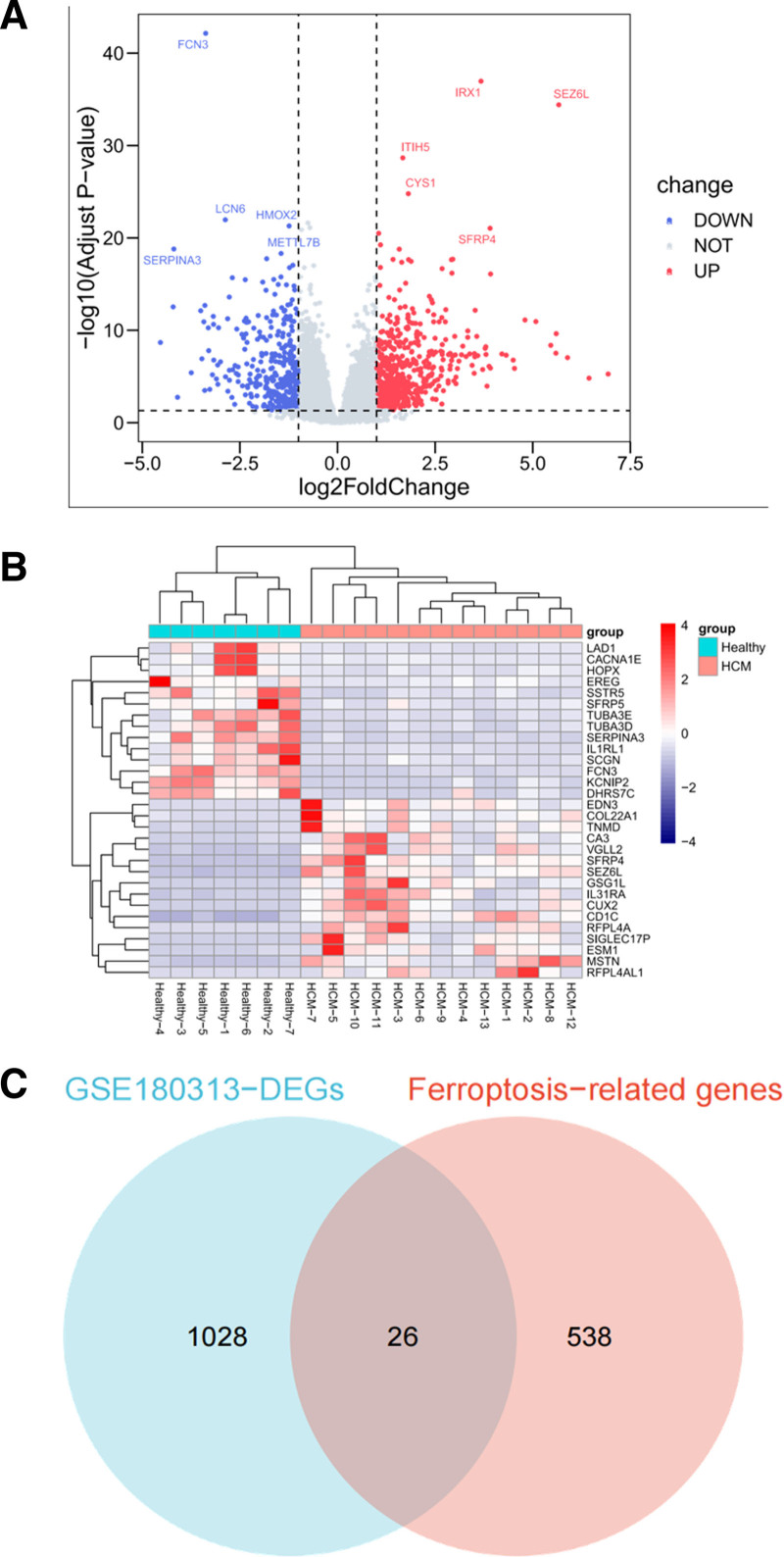
Identification of DEGs and ferroptosis-related differential genes in HCM. (A) Volcano plot showing the differential expression of genes in HCM using the GSE180313 training set. (B) Heatmap representing the expression levels of DEGs in HCM. (C) Venn diagram illustrating the overlapping genes between DEGs and ferroptosis-related genes. DEGs = differentially expressed genes, HCM = hypertrophic cardiomyopathy.

Using the thresholds of adjusted |log_2_(FC)| > 1 and *P* value < .05, a total of 1054 DEGs were obtained from the GSE180313 training set, including 692 significantly up-regulated and 362 significantly down-regulated genes (Table S2, Supplemental Digital Content, http://links.lww.com/MD/O411). Volcano plots and heatmaps were used to visualize DEGs (Fig. 2A, B). Then, combined with DEGs and SRGs, we screened 26 overlapping genes for further study (Fig. 2C).

### 3.2. Analysis of PPI networks and identification of hub genes

**Figure 3.**
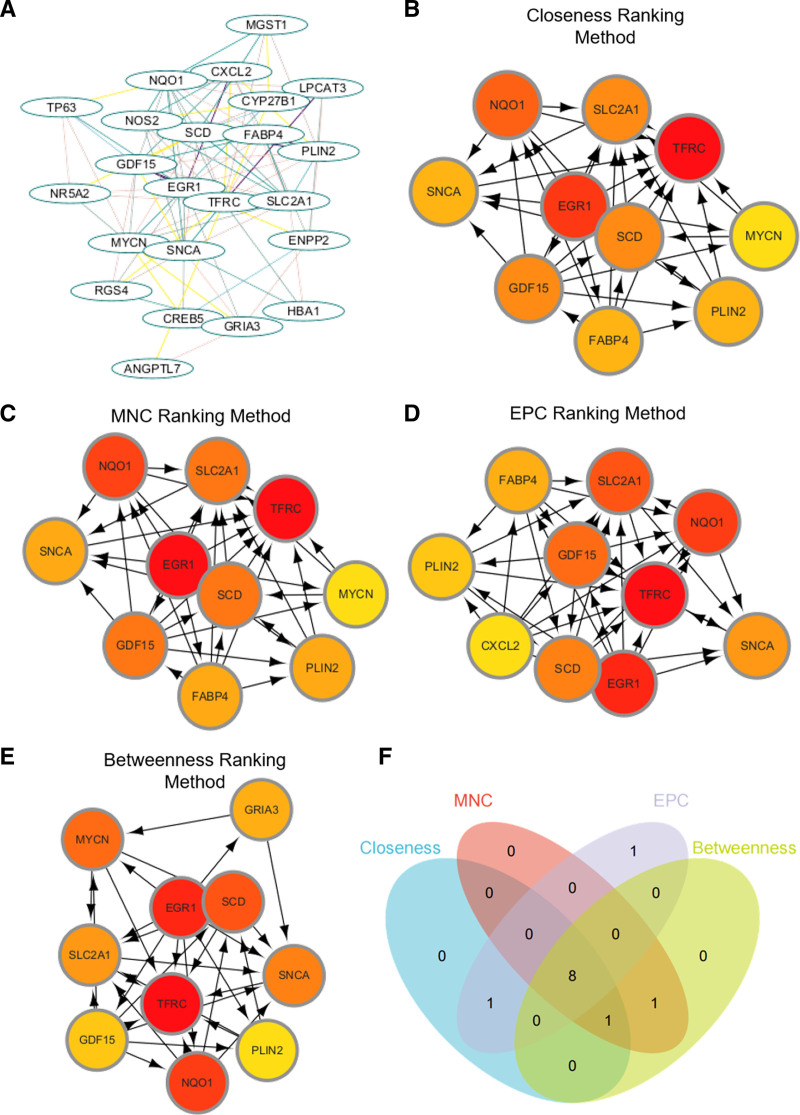
PPI network and identification of hub genes. (A) PPI network was constructed using the STRING database. (B–E) Top ten hub genes identified by different ranking approaches in cytoHubba: closeness ranking (B), maximal neighborhood component ranking (C), EPC ranking (D), and betweenness ranking (E). (F) Four common interacting hub genes identified by the intersection of the 4 ranking approaches. EPC = edge percolated component, PPI = protein–protein interaction.

**Figure 4.**
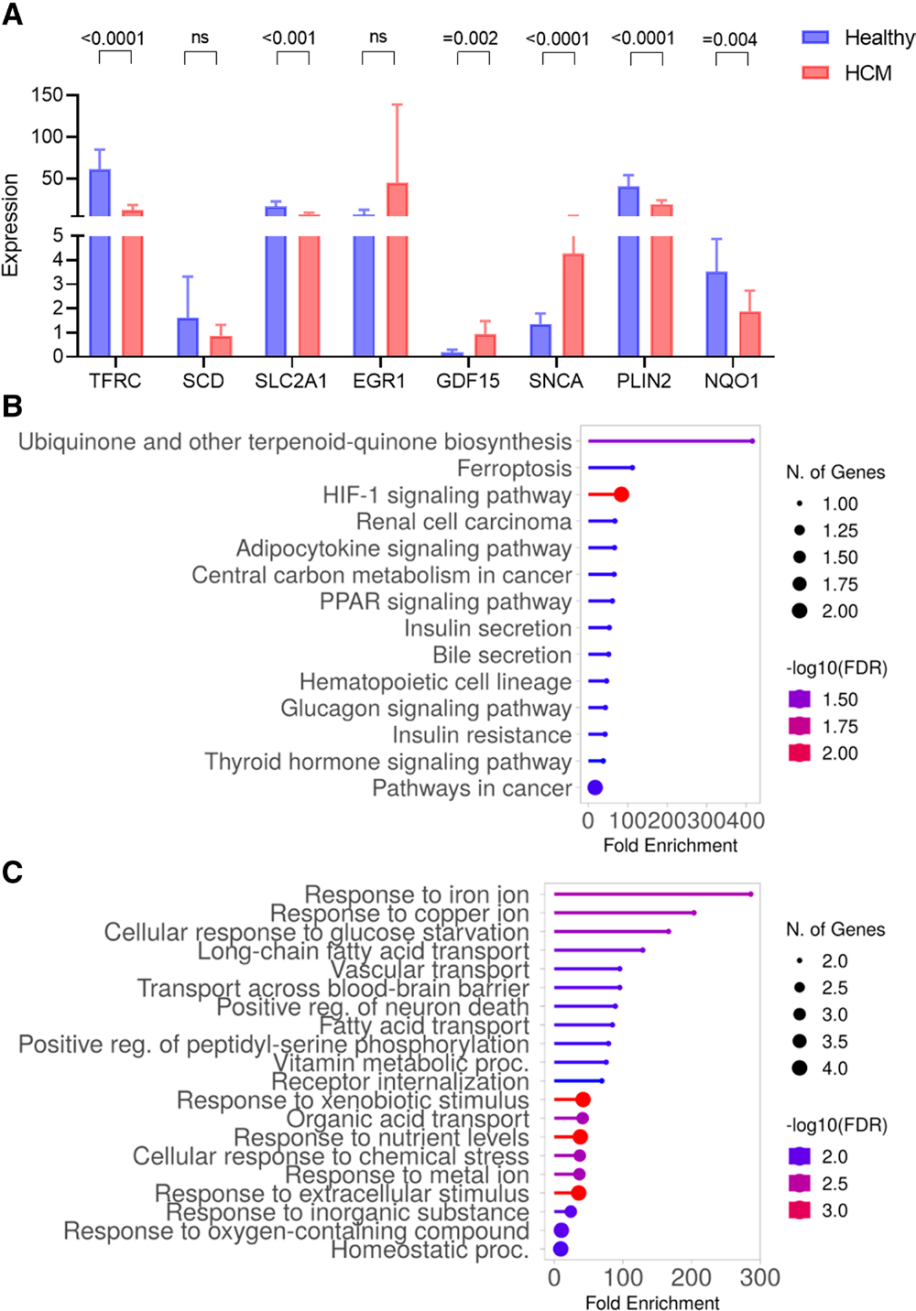
Functional enrichment analysis. (A) mRNA expression of the identified hub genes in HCM. (B) KEGG functional enrichment analysis of proteins interacting with the hub genes. (C) Gene ontology biological process analysis of proteins interacting with the hub genes. HCM = hypertrophic cardiomyopathy, KEGG = Kyoto Encyclopedia of Genes and Genomes.

The STRING database was used to build the protein–protein interaction (PPI) network of the 36 overlapped genes (Fig. 3A). Using Cytoscape v3.10.0, the resulting query protein was visualized as a PPI network, and the hub genes were identified using the cytoHubba tool, which returned a list of the top-ranked proteins. In cytoHubba, 4 ranking approaches were used to discover the hub genes: proximity ranking, maximal neighborhood component (MNC) ranking, EPC ranking, and betweenness ranking. The top ten hub genes chosen by each approach (Fig. 3B–E). TFRC, SCD, SLC2A1, EGR1, GDF15, SNCA, PLIN2 and NQO1 were discovered as 4 common interacting hub genes by the intersection of these 4 approaches (Fig. 3F). These genes mRNA expression in GSE180313 was shown in Figure 4A. After *t* test, it was found that the expression of SCD and EGR1 genes was not significantly different between HCM and healthy group. We then checked the roles of the other 6 genes through FerrDB database and found that the pro-ferroptosis gene SNCA was significantly up-regulated in HCM group, while the anti-ferroptosis genes PLIN2 and NQO1 were significantly down-regulated. The role of SLC2A1 in ferroptosis was not clear, so we selected the above 4 genes for subsequent analysis.

### 3.3. Functional enrichment analysis of 4 hub genes

According to the KEGG database, the functional enrichment analysis of proteins interacting with the 4 genes revealed a predominance of pathways involved in the Ubiquinone and other terpenoid-quinone biosynthesis, Ferroptosis, and HIF-1 signaling pathway (Fig. 4B). In GO-BP, the majority of these proteins were shown to be enriched in Response to iron ion, Response to copper ion, and Cellular response to glucose starvation (Fig. 4C).

### 3.4. Immune cell infiltration and correlation analysis in HCM

**Figure 5.**
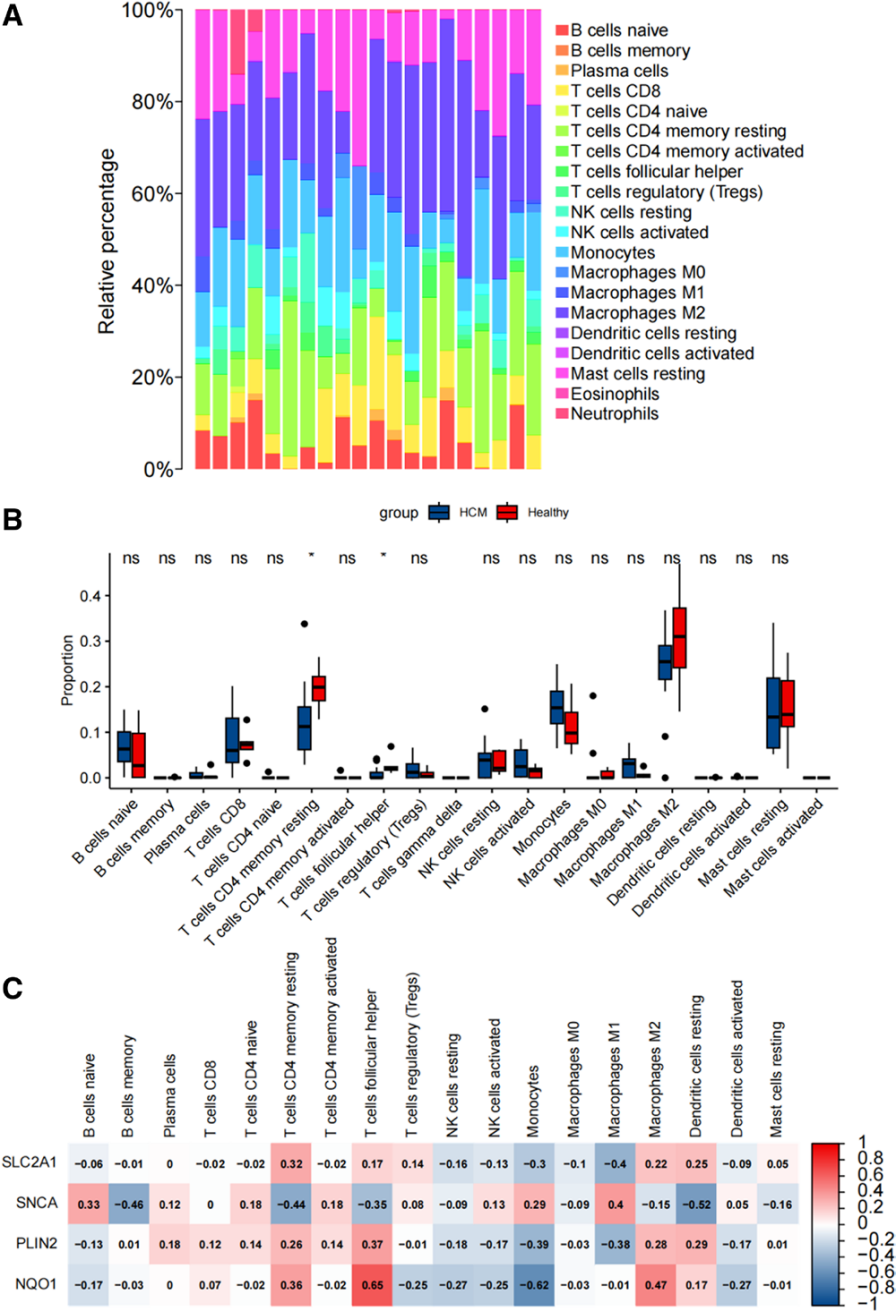
Immunological microenvironment and gene-immune cell interactions in hypertrophic cardiomyopathy. (A) Relative abundance of immune cell subtypes in HCM patients. (B) Comparison of immune cell infiltration between HCM and healthy groups. (C) Association of *SLC2A1*, *SNCA*, *PLIN2*, and *NQO1* genes with inflammatory cell infiltration. HCM = hypertrophic cardiomyopathy.

The relative abundance of numerous immune cell subtypes was determined based on the transcripts of all samples to explore the immunological microenvironment of HCM. The results of CIBERORT revealed that the most predominant infiltrative immune cells was M2 macrophages (Fig. 5A). The infiltration of immune cells in the HCM and healthy groups was evaluated. The HCM group had a lower proportion of CD4 memory resting T cells and follicular helper T cells than the Healthy group. Other immune cell subgroup proportions did not vary significantly between the 2 groups (Fig. 5B). To investigate the roles of SLC2A1, SNCA, PLIN2, and NQO1 genes in the immune microenvironment of HCM patients, we evaluated inflammatory cell infiltration. The findings showed that SNCA was adversely associated with CD4 memory resting T cells and dendritic cells. NQO1 was adversely associated with monocytes and was profitably associated with follicular helper T cells (Fig. 5C). There is a link between 4 hub genes and immune infiltration.

### 3.5. Identification and validation of diagnostic feature biomarkers

**Figure 6.**
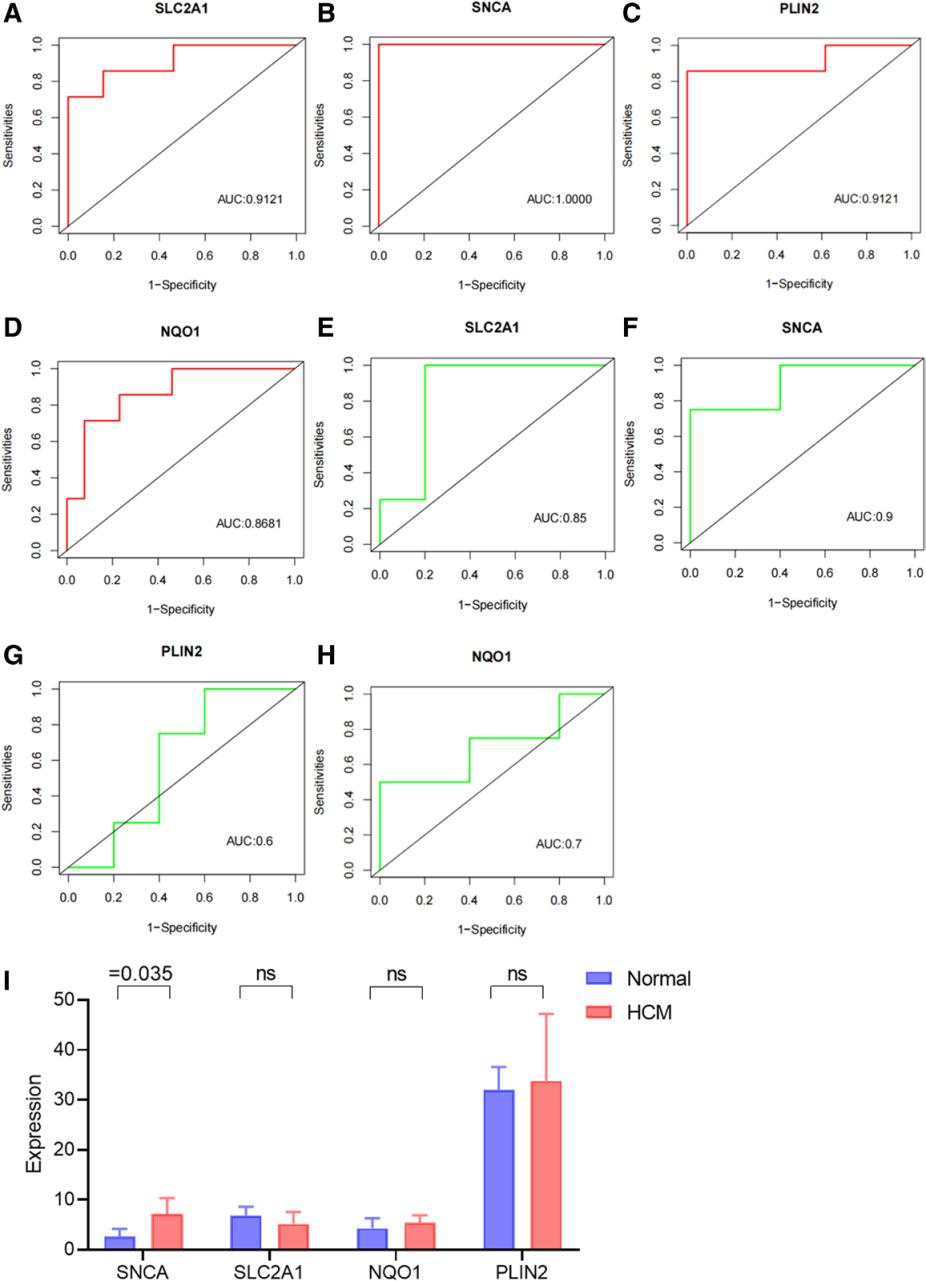
Diagnostic evaluation and gene expression levels of hub genes in hypertrophic cardiomyopathy. (A–D) Diagnostic values of the hub genes. (E–H) Gene expression levels and diagnostic values of the hub genes in the validation set. (I) Comparison of gene expression levels of the hub genes in the validation set.

ROC were used to evaluate the diagnostic value of 4 hub genes in HCM. Figure 6A to D shows the diagnostic values of the 4 hub genes in the training set GSE180313. The results showed that the AUCs of all hub genes were >0.800, among which SNCA (AUC, 1.0000) had the highest diagnostic value, followed by SLC2A1 and PLIN2 (AUC, 0.9121). In the validation set, the gene expression level and diagnostic value were further verified show the gene expression levels and diagnostic values in the GSE89714 validation set. Except PLIN2, the AUCs of all hub genes were >0.7000, of which SNCA (AUC, 0.9000) had the highest diagnostic value, followed by SLC2A1 (AUC, 0.8500; Fig. 6E–H). However, only the expression levels of SNCA were statistically significant (Fig. 6I). The results showed that the expressions of SNCA had statistical significance and high diagnostic value in HCM.

### 3.6. Upregulation of SNCA in vitro in hypertrophic cardiomyopathy

**Figure 7.**
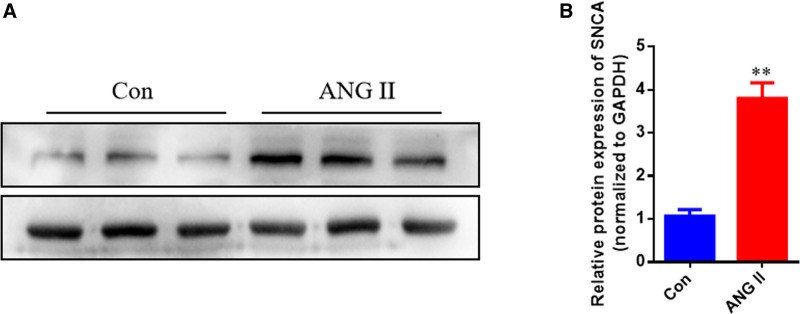
Validation of SNCA expression in hypertrophic cardiomyopathy (HCM) using an in vitro model. (A, B) Validation of SNCA expression in HCM in vitro. HCM = hypertrophic cardiomyopathy.

To validate the expression level of SNCA in HCM. We employed the H9C2 cell line as an in vitro model system and stimulated it with angiotensin II (ANG II) to induce hypertrophic cardiomyopathy-like changes. Western blot analysis was performed to assess the expression level of SNCA (Fig. 7A, B). Our findings revealed a significant upregulation of SNCA in response to ANG II stimulation in the H9C2 cell line. This observation suggests that SNCA may play a crucial role in the development and progression of HCM.

## 4. Discussion

In this study, we aimed to identify DEGs and ferroptosis-related differential genes in HCM. Our analysis revealed a total of 1054 DEGs, including 692 up-regulated and 362 down-regulated genes. By combining these DEGs with known ferroptosis-related genes, we identified 26 overlapping genes for further investigation. To gain insights into the functional relevance of these genes, we constructed a PPI network using the STRING database. From this network, we identified hub genes using the cytoHubba tool. Four common interacting hub genes were discovered: TFRC, SCD, SLC2A1, and EGR1. However, after performing *t* tests, we found that the expression of SCD and EGR1 genes was not significantly different between HCM and healthy groups. Therefore, we focused on the remaining 4 genes for subsequent analysis.

SNCA, also known as α-synuclein, drew our attention due to its known involvement in neurodegenerative disorders, such as Parkinson’s disease.^[9]^ Interestingly, recent studies have implicated SNCA in cardiovascular diseases as well. For instance, SNCA is upregulated in transitional aortic constriction mice.^[10]^ In our study, we found that SNCA was significantly up-regulated in the HCM group, suggesting a potential role in HCM pathogenesis.

On the other hand, PLIN2 and NQO1, 2 anti-ferroptosis genes, were found to be significantly down-regulated in HCM. These genes have been previously associated with oxidative stress and cell death processes. PLIN2, also known as perilipin 2, is involved in lipid metabolism and lipid droplet formation, protecting cells from excessive lipid accumulation and subsequent lipotoxicity.^[11]^ NQO1, also known as NAD(P)H quinone dehydrogenase 1, plays a crucial role in cellular defense against oxidative stress by reducing quinones and preventing their harmful effects.^[12]^

The down-regulation of PLIN2 and NQO1 in HCM suggests a dysregulation of lipid metabolism and impaired antioxidant defense mechanisms, which may contribute to the progression of the disease. These findings are consistent with the accumulating evidence implicating oxidative stress and lipid metabolism dysregulation in the pathogenesis of cardiovascular diseases, including HCM.^[13,14]^

Regarding SLC2A1, also known as glucose transporter 1 (GLUT1), its role in ferroptosis is not well-established. However, several studies have highlighted its association with metabolic reprogramming and glucose uptake in various diseases, including cancer.^[15]^ Further investigations are warranted to elucidate the potential involvement of SLC2A1 in ferroptosis regulation in the context of HCM.

To gain insights into the biological processes and pathways associated with the 4 hub genes, we performed functional enrichment analysis using the KEGG database. This analysis revealed a predominance of pathways involved in ubiquinone and other terpenoid-quinone biosynthesis, ferroptosis, and the HIF-1 signaling pathway.

The involvement of ubiquinone and terpenoid-quinone biosynthesis pathways suggests potential alterations in redox homeostasis and mitochondrial function in HCM. Previous studies have implicated mitochondrial dysfunction and oxidative stress in the pathophysiology of HCM.^[16]^ Dysregulated biosynthesis of ubiquinone and terpenoid-quinone compounds may further contribute to these abnormalities.

The enrichment of the ferroptosis pathway is particularly intriguing, given the emerging role of ferroptosis in various diseases, including cardiovascular disorders. Ferroptosis is an iron-dependent form of regulated cell death characterized by the accumulation of lipid peroxides and the disruption of redox balance.^[2]^ The dysregulation of ferroptosis-related genes, such as SNCA, PLIN2, and NQO1, suggests a potential link between ferroptosis and HCM pathogenesis.

Additionally, the enrichment of the HIF-1 signaling pathway highlights the potential involvement of hypoxia-inducible factors in HCM. Hypoxia-inducible factors play crucial roles in cellular responses to hypoxic conditions and have been implicated in cardiovascular diseases, including HCM.^[17]^ The dysregulation of this pathway may contribute to abnormal cellular responses and remodeling processes observed in HCM.

Furthermore, GO analysis revealed enrichment in response to iron ion, copper ion, and cellular response to glucose starvation. These findings provide additional evidence for the dysregulation of metal ions and metabolic processes in HCM, supporting the notion that oxidative stress and altered energy metabolism play key roles in the pathogenesis of the disease.^[18]^

To explore the immunological microenvironment of HCM, we investigated immune cell infiltration using the CIBERSORT algorithm. Our analysis revealed the presence of various immune cell subtypes in the HCM samples, with M2 macrophages being the most predominant infiltrative immune cells. M2 macrophages are known to exhibit anti-inflammatory properties and are involved in tissue repair processes.^[19]^ Their abundance suggests ongoing inflammatory and reparative processes in HCM.

Comparison of immune cell proportions between the HCM and healthy groups revealed lower proportions of CD4 memory resting T cells and follicular helper T cells in the HCM group. CD4 memory resting T cells play essential roles in immune surveillance and immunological memory, while follicular helper T cells are critical for antibody production and B cell maturation.^[20]^ The decrease in these cell subpopulations suggests alterations in immune responses and potential impairments in adaptive immunity in HCM.

Moreover, the correlation analysis between the 4 hub genes and immune infiltration provided intriguing insights into their potential roles in immune modulation. SNCA showed adverse associations with CD4 memory resting T cells and dendritic cells, suggesting its involvement in immune dysregulation in HCM. On the other hand, NQO1 exhibited adverse associations with monocytes but positive associations with follicular helper T cells, indicating its potential immunomodulatory effects in HCM.

Finally, we evaluated the diagnostic value of the 4 hub genes in HCM using ROC. Our results demonstrated promising diagnostic potential for all 4 genes, with SNCA exhibiting the highest diagnostic value (AUC, 1.0000). These findings suggest that these genes may serve as potential diagnostic biomarkers for HCM. The validation set (GSE89714) provided further support for the diagnostic value of these genes, with SNCA showing the highest AUC (0.9000) followed by SLC2A1 (0.8500). However, it is important to note that only the expression levels of SNCA were statistically significant in the validation set, highlighting the need for further validation studies with larger cohorts. Through western blot analysis, we have successfully validated the upregulation of SNCA in H9C2 cells stimulated with ANG II, confirming its potential involvement in HCM. These findings contribute to our understanding of the molecular alterations associated with HCM and provide a basis for further research aimed at developing targeted therapeutic interventions for this complex cardiovascular disorder.

This study also have some limitations. Firstly, the sample size used in this study may not be representative of the entire HCM population. The findings should be validated in larger cohorts to ensure their generalizability and reliability. Secondly, this study focused on the expression levels of 4 hub genes and their association with HCM and ferroptosis. However, the functional roles of these genes in HCM pathogenesis and ferroptosis regulation require further investigation. Additional experimental studies, such as in vitro and animal models, are needed to elucidate the underlying mechanisms.

Collectively, in future perspectives, the identification of key genes involved in ferroptosis in HCM opens up new avenues for research and potential therapeutic intervention. Future studies should focus on the functional validation of these hub genes, particularly TFRC, SLC2A1, SNCA, and PLIN2, to understand their precise roles in the pathogenesis of HCM. This could involve both in vitro and in vivo models to assess the impact of modulating these genes on disease progression and outcomes. Additionally, the exploration of the interplay between ferroptosis and immune cell infiltration in HCM is a promising area for future research. Understanding the immune landscape in HCM could lead to the development of immunomodulatory therapies that target specific cell types or pathways, potentially improving outcomes for patients. The potential for targeted therapies based on our findings is significant. For instance, drugs that can modulate the activity of the identified hub genes or interfere with the ferroptosis pathway could be developed. Preclinical studies should be conducted to test the efficacy and safety of such compounds before moving on to clinical trials. Moreover, The clinical implications of our study are manifold. Firstly, the hub genes identified could serve as biomarkers for the diagnosis and prognosis of HCM. Particularly, our findings suggest that SNCA may have the highest diagnostic value among the genes tested. Further validation in larger cohorts is warranted to establish the clinical utility of these biomarkers. Secondly, understanding the molecular mechanisms underlying HCM could lead to the development of personalized medicine approaches. By identifying patients with specific genetic profiles, we may be able to tailor treatments to their individual needs, potentially improving treatment efficacy and reducing side effects. Thirdly, the association between the hub genes and immune infiltration patterns could have implications for immunotherapies in HCM. If specific immune cells are found to play a critical role in disease progression, targeted immunotherapies could be developed to modulate their activity. Lastly, our study highlights the importance of considering ferroptosis in the management of HCM. This could lead to the inclusion of anti-ferroptotic agents in the treatment regimens for HCM patients, potentially offering a new therapeutic strategy to combat this debilitating condition.

In summary, our study identified DEGs and ferroptosis-related differential genes in HCM. We discovered 4 hub genes (SLC2A1, SNCA, PLIN2, and NQO1) with potential implications in HCM and ferroptosis. Functional enrichment analysis revealed their involvement in various biological pathways, including ferroptosis-related processes. Additionally, our findings suggested an association between these hub genes and immune infiltration in HCM. Finally, we demonstrated the diagnostic value of these genes in HCM, particularly SNCA, which showed significant diagnostic potential. This study provides valuable insights into the molecular mechanisms underlying HCM and highlights potential biomarkers for its diagnosis.

## Author contributions

**Conceptualization:** Fang Huang, Wei Chen.

**Data curation:** Fang Huang, Wei Chen, Shujuan Li.

**Formal analysis:** Shujuan Li, Ailei Zhang.

**Methodology:** Shaoqiang Zhang.

**Supervision:** Jihuai Zhao.

**Writing – original draft:** Fang Huang, Dongwei Liu.

**Writing – review & editing:** Wei Chen.

## Supplementary Material


